# Systemic Oxidative Stress and Antioxidant Capacity in Pregnant Women Exposed to Air Pollution: A Case-Control Study in Western Macedonia, Greece

**DOI:** 10.3390/cimb48060575

**Published:** 2026-05-29

**Authors:** Eirini Ilia, Dimitrios Papoutsis, Vasiliki Michou, Aikaterini Itziou

**Affiliations:** 1 Department of Midwifery, School of Health Sciences, University of Western Macedonia, 50200 Ptolemaida, Greece; dpapoutsis@uowm.gr; 2 Department of Nursing, School of Health Sciences, International Hellenic University, 57400 Thessaloniki, Greece; vmichou@ihu.gr

**Keywords:** pollution, oxidative stress, biomarkers, pregnancy

## Abstract

**Background:** Long-term exposure to ambient air pollution during pregnancy has been strongly associated with oxidative-stress-mediated adverse maternal and fetal outcomes. **Aim:** The present study aimed to evaluate systemic oxidative stress and antioxidant capacity in pregnant women residing in a highly polluted area (Kozani) compared with a less polluted region (Grevena) in Western Macedonia, Greece. **Methods:** Oxidative stress was assessed using derivatives of reactive oxygen metabolites (d-ROMs), while antioxidant capacity was evaluated through biological antioxidant potential (BAP). **Results:** The findings of the study demonstrated that pregnant women in the polluted area exhibited elevated d-ROMs levels and significantly reduced BAP levels compared with controls. Although unadjusted oxidative stress differences were not statistically significant, adjusted analyses revealed significantly higher oxidative stress in the exposed group. These results suggest that air pollution exposure is associated with systemic redox homeostasis, primarily through depletion of antioxidant defenses. **Conclusions:** This study provides novel biomonitoring evidence linking environmental exposure to redox imbalance during pregnancy. As Western Macedonia transitions to a post-lignite era, the decrease in air pollution is anticipated to lead to significant improvements in public health, while these findings establish an important baseline for evaluating the effectiveness of environmental and public health interventions.

## 1. Introduction

Air pollution derived from coal combustion and industrial activities contains a complex mixture of fly ash, heavy metals (including mercury), sulfur dioxide (SO2), nitrogen oxides (NOx), and particulate matter (PM). These contaminants have a significant impact on human health, increasing the risk of disease and early mortality [1]. Sichletidis et al. [2] investigated the negative effects of air pollution on children’s respiratory system in Western Macedonia and found that schoolchildren in Ptolemaida, a city in the Kozani prefecture, a highly polluted city, had higher rates of rhinitis and infectious bronchitis than in Grevena, a less industrialized and less polluted region. Furthermore, Spyratos et al. [3] found a correlation between air pollution and chronic nasal symptoms and nasal blockage in a 19-year cohort study of children in Ptolemaida (Kozani prefecture, Western Macedonia). It has been proposed that the negative impact of PM2.5 on chronic obstructive pulmonary disease functions by reducing patients’ antioxidant potential [4]. Even at extremely low quantities, non-essential heavy metals (Hg, Cd, Pb, etc.) can be harmful to health [5]. At a molecular level, their harmful effects on the body are mostly caused by disruptions in intracellular metabolic systems, damage to DNA, oxidative lipid/protein degradation brought on by elevated oxidative stress, mitochondrial damage induction, and programmed cell death [6].

Vulnerable populations, such as pregnant women, have an elevated risk profile. During pregnancy, oxidative stress balance is particularly delicate. Elevated oxidative stress has been associated with complications such as gestational diabetes and fetal growth restriction [7,8]. Toescu et al. [9] highlighted that normal pregnancy involves increased oxidative stress, necessitating robust antioxidant defenses to maintain maternal and fetal health. Exposure to air pollution can impact the growing fetus as well as the mother. Birth outcomes like low birth weight, early delivery, and intrauterine growth retardation (IUGR) have been linked to pregnant women’s exposure to air pollution [10]. It is unclear exactly which molecular pathway results in the health effects linked to air pollution. Oxidative stress has been linked to several unfavorable birth and health outcomes related to exposure to air pollution [11]. Jia et al. [12] reported that augmented PM2.5 levels increased preeclampsia risk, while Pham et al. [13] supported that environmental factors may induce augmented maternal oxidative stress and thus child mental disorders. Zadorozhnaja et al. [14] found high values of arsenic, cadmium, copper, lead, mercury, and zinc in placenta from 200 pregnant women living in urban areas of Ukraine, which could play an important role in reproductive disorders. Because of their increased energy consumption and changed physiological processes, pregnant women are more vulnerable. Oxidative stress in pregnancy can affect the mother’s health and hinder fetal development via malnutrition and inadequate placental blood flow [15] and may lead to preeclampsia or preterm labor [16]. Moreover, research has indicated that even normal pregnancy can trigger a condition of mild oxidative stress and result in lipid peroxidation [17].

Therefore, the present study aimed to investigate systemic oxidative stress and antioxidant capacity in pregnant women exposed to differing levels of air pollution, providing mechanistic insights and supporting the development of targeted public health strategies.

## 2. Materials and Methods

### 2.1. Sample Size Estimation

A priori power analysis was conducted using G*Power (version 3.1, Germany) to determine the minimum sample size required to detect differences in oxidative stress and antioxidant capacity between pregnant women residing in high- and low-pollution areas. The primary outcomes of interest were serum d-ROMs levels and BAP values. Assuming a moderate effect size (Cohen’s d = 0.50), a two-tailed significance level of α = 0.05, and a statistical power of 0.80, the minimum required sample size for an independent samples comparison (two groups) was 64 participants (32 per group). To ensure sufficient statistical power and account for potential variability in biological measures, a total sample of at least 80 participants was considered adequate. The final study sample included 100 participants (50 per group), exceeding the minimum required sample size and thereby providing sufficient power to detect meaningful differences and to support adjusted analyses, including ANCOVA models controlling for potential confounding variables.

### 2.2. Participants and Study Design

For this case-control study, a total of 100 pregnant women were recruited between March 2024 and March 2025. Fifty pregnant women were residents of a highly polluted area in the heavily industrialized prefecture of Kozani (Western Macedonia), where four lignite power stations are located in the Eordea basin in the axis of the Greek cities of Kozani and Ptolemaida, producing more than 70% of the country’s power requirements.

Fifty pregnant women, serving as controls, were residents of the prefecture of Grevena (Western Macedonia), an area with no lignite power stations and lower air pollutant levels, and were also recruited into the study. Exposure to air pollution was based on mean annual air pollutant concentrations and meteorological data from the National Air Pollution Monitoring Network in the studied areas, as defined by Begou et al. [18], calculated using the Air Quality Health Index (AQHI). In fact, the number of days in the category of “high” health risk was 7 in Kozani, while 0 days were categorized as “high” risk in Geneva. This is also in accordance with the results based on the dataset from Air Quality Monitoring Stations in Western Macedonia, Evagelopoulos et al. [19], and Evagelopoulos et al. [20]. Moreover, according to the IQAir World’s Most Polluted Cities 2024 Report and the IQAir World’s Most Polluted Cities 2025 Report, PM2.5 concentrations in the study region show a consistent spatial gradient, with higher levels observed in the Kozani–Ptolemaida area compared with Grevena. This difference indicates a relatively higher pollution burden in the Kozani–Ptolemaida urban/industrial zone, which is in line with the increased number of “high” AQHI risk days observed in Kozani. These contemporaneous data support both the exposure classification and the observed inter-regional differences in air quality within the study period.

Participants were recruited from public hospitals of Kozani–Ptolemaida and Grevena, respectively. Coordination was conducted with obstetricians and prenatal nurses, who helped identify eligible participants. Flyers and brochures, as well as information sheets with the study’s purpose, eligibility, and contact information, were placed in prenatal waiting areas. A trained research assistant approached women in the waiting area or after consultations, provided an information sheet, and explained the study. Women who expressed interest were screened for eligibility, and those who consented were enrolled. Each enrolled participant was scheduled for a blood collection appointment at the Laboratory of Clinical Biochemistry, University of Western Macedonia. Transportation support was provided if needed. Eight-hour fasting blood samples of all women were collected with a microvette; the serum was collected after centrifugation for 90 s at 6000 rpm and analyzed immediately. The age range of pregnant women was 21–40 years. Pregnant women were in the third trimester of pregnancy and resided in the given geographical location for the full duration of their pregnancy. To avoid misclassification of exposure due to a change in maternal residence, we recruited participants who had not changed residence in the previous year. Participants with known chronic diseases, medication use, or pregnancy complications were excluded, and all subjects were recruited from similar socioeconomic and demographic backgrounds within the study regions. In addition, active smoking during pregnancy was an exclusion criterion. The volunteer study participants completed a pre-designed interviewer-administered questionnaire. The questionnaire provided residence time, demographic characteristics, pregnancy-related information, etc. They were informed about the purpose of the study, and informed consent was obtained from each participant. The study was conducted in accordance with the ethical standards laid down in the 1964 Declaration of Helsinki and its later amendments [21]. Ethical approval for this study was granted by the Institutional Research Ethics Committee at the University of Western Macedonia (Protocol Approval Reference: 94/29 August 2022). Exclusion criteria included hypertension, diabetes, placenta pervia, genital tract infections, and multiple pregnancies.

### 2.3. Derivatives of Reactive Oxygen Metabolites (d-ROMs)

A d-ROMs test was created by Carratelli and his colleagues in Italy to conduct a thorough assessment of oxidative stress. This test has made it possible to quickly and accurately assess oxidative stress, which has aided in the completion of cutting-edge clinical research. The d-ROMs test measures hydroperoxide blood levels, a metabolite of active oxygen and/or free radicals, using a color reaction method. It also provides a comprehensive assessment of the in vivo state of oxidative stress levels. In the d-ROMs test, measured using a commercial assay kit (Diacron International, Grosseto, Italy), an acidic buffer (reagent R2) releases iron from plasma protein, which causes hydroperoxide in a blood sample to produce alkoxyl and peroxyl radicals via the Fenton reaction. These radicals produce pink-colored derivatives by progressively oxidizing colorless alkyl-substituted aromatic amines dissolved in the dye mixture (reagent R1). It took 5 min to measure this pink reaction with a photometer (505 nm). The d-ROMs test result was expressed in U.CARR, or Carratelli units. 1 U.CARR is equivalent to 0.08 mg/dL of water with hydrogen peroxide [22].

### 2.4. Biological Antioxidant Potential (BAP)

Using the BAP test created by Carratelli and his colleagues in Italy, the antioxidant power of was assessed using a commercial assay kit (Diacron International, Grosseto, Italy). To assess antioxidant power, the BAP test measures the samples’ capacity for reduction, or the ability to change trivalent iron (Fe^3+^) into bivalent iron (Fe^2+^). The BAP test measures the antioxidant power of biological fluid samples and foods by applying the same chemistry principle as the Ferric Reducing Antioxidant Power (FRAP) assay. A cuvette containing a solution of thiocyanate derivative (reagent R1) was filled with 50 μL of trivalent iron salt (FeCl_3_: reagent R2). A photometer was used to measure the reagent that was reddened by trivalent iron ions (Fe^3+^). Following the addition of 10 μL of plasma to the cuvette, the changes were monitored once more for 5 min at a wavelength of 505 nm, during which time the antioxidants in the plasma reduced the trivalent irons to bivalent irons (Fe^2+^) and caused them to become decolored. One of two-unit expressions was provided: μmol/L or μEq/L [23].

### 2.5. Statistical Analysis

Data on serum d-ROMs (reactive oxygen metabolites) and serum BAP (biological antioxidant potential) levels in a sample from the Kozani prefecture and a control group from Grevena (Western Macedonia) were tested using nonparametric statistics (Mann–Whitney U test, *p* < 0.05). The goal was to determine whether there was a significant difference between the sample and control group for each of these parameters. The results from the Mann–Whitney U test for both parameters were examined to assess any statistically significant variations between the groups, providing insights into potential differences in d-ROMs and BAP between the two locations. To examine potential confounding variables, an analysis of covariance (ANCOVA) was performed with d-ROMs and BAP values as dependent variables, region of residence as a fixed factor, and body mass index (BMI), years of residence in the region, gestational age, weight at the start of pregnancy, and current weight at the time of participation as covariates. Levene’s test was applied to assess the homogeneity of variances. Adjusted means were calculated to assess regional differences after controlling for covariates. Spearman’s rank correlation coefficient was used to assess associations between oxidative stress biomarkers (d-ROMs and BAP) and clinical variables, including gestational age and years of residence. Statistical significance was set at *p* < 0.05 (two-tailed). Statistical analysis was performed using IBM SPSS Statistics 19.0.

## 3. Results

### 3.1. Participants’ Characteristics

All descriptive characteristics of the participants are presented in Table 1.

### 3.2. Derivatives of Reactive Oxygen Metabolites (d-ROMs)

**Figure 1:**
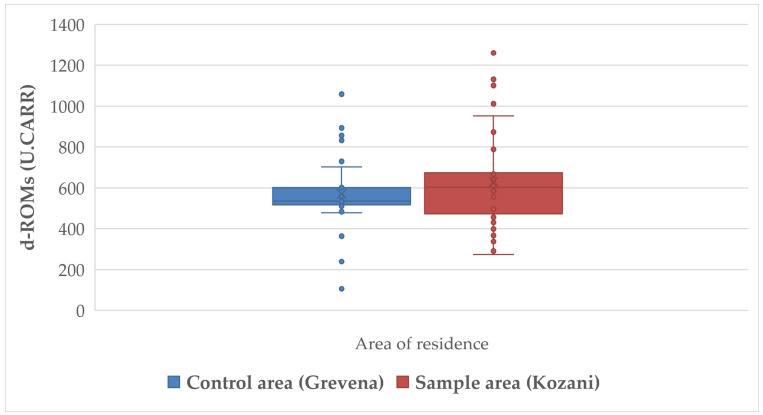
Maternal serum d-ROMs levels (U.CARR) according to the area of residence. The boxplots illustrate the median line, interquartile range (IQR), and mean value (x); individual inner points represent the exact distribution of the 100 participants (n = 50 per group).

The serum oxidative stress levels (d-ROMs levels) were 630.22 ± 33.76 U.CARR in pregnant women living in the Kozani prefecture (sample area) and 569.62 ± 22.52 U.CARR in pregnant women living in Grevena (control area) (Figure 1). The nonparametric Mann–Whitney U test was used to compare d-ROMs levels in serum between the two regions (Kozani prefecture and Grevena prefecture). The results showed that although d-ROMs levels were higher in the sample group compared with the control, there was no statistically significant difference between the two groups (*p* = 0.295) (Table 2).

The adjusted mean values of d-ROMs by region showed that pregnant women from the control area had lower levels of d-ROMs (559.12 ± 26.75 U.CARR, 95% CI: 506.01–612.23) compared with women from the sample area (640.72 ± 26.75 U.CARR, 95% CI: 587.61–693.83) after adjusting for BMI, years of residence in the region, gestational age, weight at the beginning of pregnancy, and current weight at the time of participation. After controlling for potential confounding factors, women from the control area showed lower oxidative stress compared with women from the sample area. The comparison of the adjusted mean values of d-ROMs between regions showed a statistically significant difference in pregnant women from the control area, who had lower levels of d-ROMs compared with women from the sample area (mean difference = −81.60, SE = 38.21, *p* = 0.035, 95% CI: −157.48 to −5.73), suggesting lower oxidative stress in the control group after adjusting for confounding factors.

### 3.3. Biological Antioxidant Potential (BAP)

**Figure 2:**
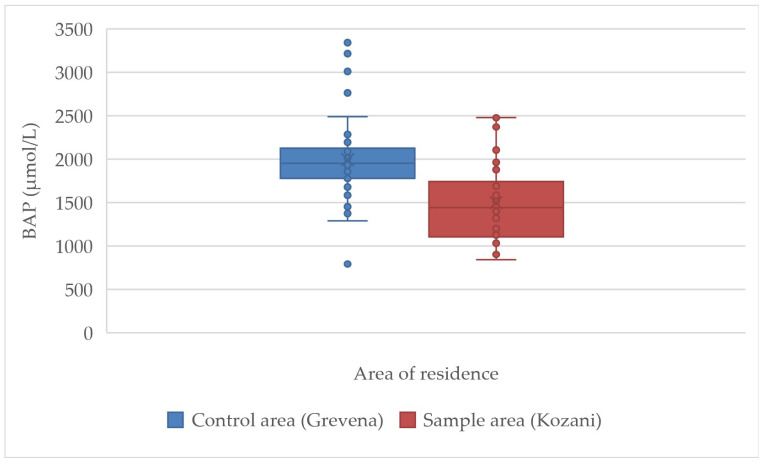
Maternal serum biological antioxidant potential (BAP) levels (μmol/L) according to the area of residence. The boxplots illustrate the median line, interquartile range (IQR), and mean value (x); individual inner points represent the exact distribution of the 100 participants (n = 50 per group).

The serum biological antioxidant potential levels (BAP levels) were 1500.18 ± 60.38 μmol/L in pregnant women living in the Kozani prefecture (sample area) and 1996.54 ± 68.87 μmol/L in pregnant women living in Grevena (control area) (Figure 2). Given the non-normal distribution of the data, the nonparametric Mann–Whitney U test was applied to compare the levels of the BAP parameter between the two regions (Kozani prefecture and Grevena prefecture). The analysis showed that there was a statistically significant difference between the two groups (*p* = 0.000), suggesting that BAP values differ substantially between the regions. In particular, the Grevena group showed higher BAP levels compared with the Kozani prefecture group (mean rankings: 49.5 vs. 30.0) (Table 3).

After adjusting for potential confounding variables (body mass index, years of residence in the area, gestational age, weight at the beginning of pregnancy and current weight at participation), analysis of covariance (ANCOVA) confirmed the existence of a statistically significant effect of region on BAP (F(1.93) = 31.03, *p* < 0.001, partial η^2^ = 0.250), indicating higher antioxidant capacity in Grevena regardless of confounding factors. The estimated marginal means showed that women from the control area had a mean BAP of 1996.29 (SE = 62.31, 95% CI: 1872.56–2120.01), while women from the sample area had a mean of 1500.43 (SE = 62.31, 95% CI: 1376.71–1624.16). Pairwise comparisons confirmed that the difference between the two regions was statistically significant, with a mean difference of 495.85 units (95% CI: 319.10–672.61, *p* < 0.001) (Table 4).

### 3.4. Association Between Residence Duration and Oxidative Stress Markers

**Figure 3:**
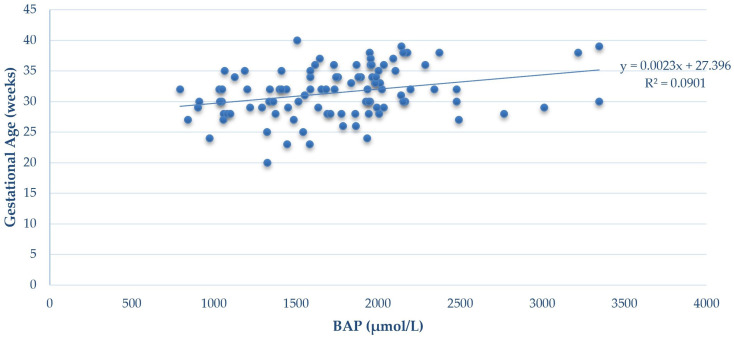
Correlation between gestational age and maternal serum biological antioxidant potential (BAP) levels. The scatter plot illustrates the positive correlation between the weeks of gestation and BAP concentrations (μmol/Λ) across the study population. The solid line indicates the linear regression trendline accompanied by its 95% confidence interval bands. The embedded metrics represent the Spearman rank correlation coefficient (r) and the corresponding statistical significance level (*p*).

No significant correlations were found between years of residence and d-ROMs (r = 0.114, *p* = 0.258) or BAP levels (r = 0.002, *p* = 0.982). This indicates that the length of residence alone may not adequately reflect an individual’s exposure to air pollution or their biological susceptibility to oxidative stress during pregnancy. On the other hand, a correlation analysis revealed a significant positive association between gestational age and BAP levels (r = 0.323, *p* = 0.001) (Figure 3), suggesting that antioxidant capacity increases as gestation progresses. However, no significant correlation was found between gestational age and d-ROMs levels (r = 0.088, *p* = 0.384).

## 4. Discussion

This study provides evidence that air pollution exposure during pregnancy may contribute to changes in oxidative stress markers, primarily through a reduction in antioxidant capacity rather than overt elevation of oxidative markers alone. This finding is particularly important, as it suggests that antioxidant depletion may precede detectable oxidative damage, representing an early biomarker of environmental stress. This study adds to the limited evidence assessing both oxidative stress and antioxidant capacity in pregnant populations exposed to differing air pollution levels in these regions. The results revealed augmented levels of systemic oxidative stress in the serum of pregnant women living in the highly polluted area compared with the controls. The serum oxidative stress levels in both groups (d-ROMs levels) 630.22 ± 33.764 (Kozani prefecture—sample area) and 569.62 ± 22.519 (Grevena—control area) U.CARR were higher than the standard value (250–300 U.CARR) as derived from manufacturer guidelines, where this range is generally considered indicative of a normal oxidative stress. It should be reported that even if a woman has no problems by the end of her pregnancy, d-ROMs levels are known to rise in comparison to the nonpregnancy time [24,25]. Previous studies on maternal plasma have reported augmented d-ROMs levels in the early puerperium, which gradually decreased in the postpartum period but even remained elevated 3 months after childbirth [26]. It is important to note that oxidative stress is physiologically elevated during pregnancy [27], which may partly explain the elevated d-ROMs values observed in both groups; that is why pregnancy should be treated using oxidative stress prevention techniques, such as researching the benefits of folic acid supplements and the adverse effects of iron supplementation [28]. However, the significantly higher levels in the polluted area suggest an additional environmental contribution.

Our findings showed that d-ROMs levels were higher in pregnant women living in the polluted area, 630.22 ± 33.764 U.CARR, compared with the controls, 569.62 ± 22.519 U.CARR. Notably, adjusting for maternal baseline characteristics in our multivariable ANCOVA model accounted for underlying error variance. It revealed a statistically significant increase in d-ROMs levels among residents of the polluted area (*p* = 0.035 vs. unadjusted *p* = 0.295). This effect remained directionally consistent across both analytical approaches but should be replicated in larger sample sizes to rule out residual confounding. Our results are in compliance with previous studies revealing significantly augmented values of oxidative stress biomarkers in pregnant women living in polluted areas compared to control areas. Notably, Nagiah et al. [29] estimated increased DNA damage assessed with the comet assay and malondialdehyde (MDA) levels in pregnant women in the third trimester living in an industrialized area compared with controls. Our findings align with previous research that supports the impact of air pollution on systemic oxidative stress in pregnant women [30]. At a molecular level, our findings should be interpreted with caution, as the biomarkers used in this study (d-ROMs and BAP) reflect overall oxidative status rather than specific sources or pathways of reactive oxygen species (ROS) generation. Therefore, although higher exposure to air pollutants was associated with alterations in systemic redox balance, no direct mechanistic conclusions regarding ROS origin or mitochondrial involvement can be drawn. Moreover, human exposure to air pollution is known to increase oxidative stress as measured by MDA levels [31]. Comparable findings were reported by Wang et al. [32] in research conducted with 305 pregnant individuals in western New York that investigated the link between exposure to PM2.5, NO_2_, and PAHs and urinary oxidative stress biomarkers, such as MDA and 8-hydroxy-2′-deoxyguanosine (8-OHdG).

Moreover, previous studies support the association between air pollution exposure during pregnancy and increased oxidative stress, particularly in adverse outcomes such as preeclampsia. For instance, Juan-Reyes [33] reported elevated oxidative stress markers in preeclamptic women exposed to higher pollution levels during early pregnancy. This is consistent with evidence that increased reactive oxygen species (ROS) can promote endothelial dysfunction through neutrophil activation, a key mechanism in preeclampsia development [34]. In line with this, earlier studies have found a significant association between prenatal exposure to PM2.5 in ambient air pollution and an increased risk of preeclampsia, which is believed to be connected to oxidative stress in the placenta, as depicted by the oxidative stress biomarker 3-nitrotyrosine [35]. Increased oxidative stress has also been linked to fetal growth-related complications. Elevated levels of lipid peroxidation markers, such as malondialdehyde (MDA), have been observed in pregnancies complicated by intrauterine growth restriction (IUGR), reflecting placental and endothelial damage [36]. Furthermore, environmental exposures, including air pollutants and arsenic, may exacerbate oxidative imbalance by disrupting antioxidant systems, such as folate metabolism, thereby contributing to impaired fetal growth [37,38]. Additional evidence suggests that pollution-induced oxidative stress may contribute to early pregnancy loss and preterm birth through mechanisms involving systemic inflammation and oxidative DNA damage [39,40]. Biomarkers such as 8-OHdG and increased xanthine oxidase activity further support the role of oxidative damage and endothelial dysfunction in these outcomes [40,41]. Overall, these findings reinforce the relevance of oxidative stress as a key biological pathway linking environmental pollution exposure to adverse maternal and neonatal outcomes, supporting the patterns observed in the present study.

The results of the current investigation showed lower levels of systemic biological antioxidant potential in the serum of pregnant women living in the highly polluted area compared with the controls. The serum biological antioxidant potential levels in both groups (BAP levels), 1500.18 ± 60.382 (Kozani prefecture—sample area) and 1996.54 ± 68.872 (Grevena—control area) μmol/L, were lower than the optimum value (>2200 μmol/L). According to previous studies using the BAP assay and manufacturer interpretative criteria, antioxidant capacity in normal serum is generally considered adequate when BAP values exceed 2200 μmol/L, particularly in healthy adults [23]. Furthermore, studies assessing oxidative balance during pregnancy and neonatal circulation have reported BAP values above 2200 μmol/L in umbilical cord blood, suggesting relatively strong antioxidant potential during the perinatal period [42]. Additionally, Fukase et al. [43] found that median maternal BAP values decreased from 2289 μmol/L at 12 weeks of gestation to 1768 μmol/L at 24 weeks and 1654 μmol/L at 36 weeks. In contrast, umbilical cord venous blood BAP values were significantly higher at delivery (2461 μmol/L). In the same study, oxidative stress levels were significantly lower and antioxidant capacity significantly higher in cord blood compared with maternal blood, suggesting enhanced fetal antioxidant protection. Therefore, the reduced BAP levels observed in both study groups in the present study may indicate decreased antioxidant capacity during the third trimester of pregnancy. However, these findings should be interpreted cautiously, as pregnancy-specific reference ranges for BAP have not been firmly established, and physiological oxidative stress changes occur throughout gestation and around delivery.

In fact, antioxidant capacity during pregnancy is quite complex. A significant positive correlation was observed between gestational age and BAP levels, suggesting that antioxidant capacity may change during pregnancy progression. Previous studies have shown that oxidative stress increases during gestation, with maternal d-ROMs increasing and BAP decreasing between 16 and 30 weeks of pregnancy, reflecting an increase in oxidative burden [44]. However, oxidative stress levels after 36 weeks of gestation appear to remain relatively stable until delivery [43]. The positive association between gestational age and BAP levels observed in the present study may reflect a compensatory antioxidant response during late pregnancy, although this interpretation remains speculative given the non-specific nature of the marker. Similar findings were reported by Yuba et al. [45], who observed elevated d-ROMs values accompanied by decreased BAP levels in pregnant women near delivery, indicating increased oxidative stress and reduced antioxidant capacity during late gestation.

Previous investigations have shown that pregnancy is associated with altered antioxidant defense and increased oxidative stress, although findings on specific enzyme activity remain inconsistent. Some reports describe increased antioxidant enzyme activity, while others show reduced levels, likely reflecting the dynamic balance between oxidative load and antioxidant depletion under sustained stress conditions [46,47,48]. Persistent oxidative stress may impair antioxidant defenses by increasing enzyme turnover and inhibiting regulatory pathways such as Nrf2, ultimately reducing the body’s capacity to counteract reactive oxygen species [49,50]. In the present study, the significantly lower biological antioxidant potential (BAP) observed in pregnant women residing in highly polluted areas supports the hypothesis that environmental exposure contributes to antioxidant depletion. This finding is consistent with previous observations of reduced antioxidant capacity and altered oxidative stress markers in populations exposed to higher pollution levels [29].

Reduced antioxidant capacity during pregnancy has been associated with adverse outcomes, particularly in conditions linked to increased oxidative stress. For example, decreased antioxidant status and glutathione-related activity have been reported in preterm births, reflecting an impaired ability to neutralize oxidative damage [51,52,53,54]. These findings suggest that environmental factors that exacerbate oxidative stress may further compromise maternal antioxidant defenses, with potential implications for pregnancy outcomes. Research by You et al. [55] further suggests that, at a molecular level, high PM2.5 exposure in mothers and infants may impair mitochondrial oxidative function in newborns, potentially contributing to health complications. A pilot study investigating women with preeclampsia exposed to air pollution found significant correlations between increased oxidative stress markers and neonatal diseases such as intrauterine growth restriction (IUGR) and necrotizing enterocolitis (NEC). This underscores the potential for environmental pollutants to contribute to adverse neonatal health outcomes through oxidative stress mechanisms. However, a study by Zhang et al. [56] measured increased antioxidant response during the first trimester in pregnant women’s serum exposed to PM2.5. It is possible that the distinct characteristics of pregnant women could influence how they react to exposure-induced oxidative stress levels [57]. Overall, the observed reduction in antioxidant capacity in polluted environments reinforces the role of air pollution as a contributing factor to oxidative imbalance during pregnancy, in line with the findings of the present study.

Importantly, the impact of air pollution may be more pronounced in individuals with increased biological susceptibility or higher exposure levels. Evidence linking prenatal exposure to air pollution with long-term outcomes, such as childhood asthma and cancer, further underscores the significance of this issue [58]. A deeper comprehension of how PM2.5 modulates antioxidant capacity during pregnancy could offer crucial insights into the pathological underlying mechanisms. Although air pollution influences individuals across all areas, age groups, and social classes, it is probable that those who are more susceptible due to high exposures to contaminated air or have increased biological vulnerability will experience more serious health effects [58]. Discoveries indicating that certain antioxidation genotypes may alter the harmful health impacts of pollution [59] encourage additional research into the influence of antioxidant gene polymorphisms in heightening vulnerability to adverse pregnancy outcomes due to pollution.

No significant correlations were observed between years of residence and d-ROMs or BAP levels. This finding suggests that the duration of residence alone may not adequately reflect an individual’s exposure burden or biological susceptibility to air-pollution-related oxidative stress during pregnancy. Oxidative stress responses are likely influenced by multiple interacting factors, including pollutant concentration, temporal variations in exposure, occupational and indoor environmental conditions, lifestyle characteristics, and individual biological susceptibility. Furthermore, residence duration may serve as an imprecise proxy for cumulative exposure, particularly in environmental health studies where personal exposure patterns and spatial variability in air pollution levels are not directly assessed. Similar methodological limitations have been discussed in previous epidemiological studies investigating air pollution exposure during pregnancy and its association with adverse health outcomes, underscoring the need for more refined exposure assessment and biomonitoring strategies [32].

A limitation of the study is the lack of personal exposure measurements and detailed time–activity data. Therefore, our findings should be interpreted as associations at the group level rather than precise individual-level exposure–response relationships. Importantly, despite this limitation, the observed differences in oxidative stress (d-ROMs) and antioxidant capacity (BAP) between groups were statistically significant and biologically plausible, supporting ambient air pollution as a contributing factor. Another limitation is that detailed quantitative data on dietary intake, antioxidant supplementation, passive smoking exposure, occupational exposure, indoor pollution sources, and traffic-related exposure were not systematically collected; therefore, comprehensive adjustment for all potential lifestyle and environment-related confounders was not possible. Consequently, the observed associations may, in part, be influenced by unmeasured confounding factors. In addition, although all participants were recruited during the third trimester of pregnancy, a significant difference in gestational age was observed between groups. Since oxidative stress biomarkers vary physiologically throughout gestation, this difference may have influenced biomarker levels to some extent. However, gestational age was included as a covariate in the adjusted analyses to minimize its potential confounding effect. Yet, given the clear environmental contrast between the study areas and the consistency of our findings with existing literature, we believe that ambient air pollution remains a biologically plausible contributing factor to the observed differences in oxidative stress and antioxidant capacity.

A key strength of this study is the use of clinically applicable and widely used biomarkers (d-ROMs and BAP), providing translational relevance for the assessment of systemic oxidative status in population-based settings. Furthermore, this study offers baseline data in a region undergoing a transition away from lignite-based energy production, highlighting its importance for future environmental health assessments.

## 5. Conclusions

Overall, oxidative stress plays a crucial role in the reproductive health of females, with environmental pollution acting as a significant contributor to the formation of reactive oxygen species. Exposure to air pollution during pregnancy is associated with impaired antioxidant capacity and altered systemic redox balance. While oxidative stress levels may not always show significant elevation, the depletion of antioxidant defenses represents a warning sign and a critical indicator of increased biological vulnerability. It suggests that pollution still has a biologically meaningful, potentially harmful effect on health. This lower antioxidant capacity makes the body more vulnerable. If pollution persists or worsens, oxidative stress could eventually rise significantly or cause more damage over time. Maintaining a balance between oxidants and antioxidants is essential for maternal and fetal well-being, yet environmental pollution disrupts this equilibrium. These findings support the use of d-ROMs and BAP as complementary biomarkers for assessing environmental exposure effects during pregnancy. Given the limited specificity of the biomarkers used, these findings should be interpreted as indicative of general oxidative imbalance rather than evidence of specific underlying biological mechanisms. Further large-scale and longitudinal studies are required to confirm these results and elucidate causal mechanisms. Given the profound impact of oxidative stress on pregnancy, it is imperative to implement preventive measures before and during gestation. Identifying critical periods of exposure, recognizing at-risk newborns, and promoting targeted interventions are vital steps in mitigating the effects of pollution-related oxidative stress. Additionally, policies aimed at reducing air pollution and raising awareness among pregnant women are necessary to safeguard maternal and fetal health.

## Figures and Tables

**Table 1:** Descriptive characteristics of pregnant women.

Groups	Control Area (n_A_ = 50)		Sample Area (n_B_ = 50)	
Maternal age (year group)	18–23	4	18–23	2
	24–29	4	24–29	7
	30–35	34	30–35	24
	36–40	8	36–40	17
Maternal weight at the beginning of pregnancy (kg)	76.54 ± 10.17		69.04 ± 10.50	
Maternal weight at test (kg)	86.32 ± 10.05		77.88 ± 10.86	
Height (m)	1.69 ± 0.05		1.68 ± 0.06	
Gestational age at test (week)	34.88 ± 2.91		31.02 ± 3.76	
Residence time (years)	20.94 ± 13.82		21.32 ± 11.55	

Note: Data are expressed as frequencies (v) and as mean ± standard deviation.

**Table 2:** Comparison of serum d-ROMs levels between pregnant women in control (Grevena) and polluted (Kozani) areas using the Mann–Whitney U test.

Parameter	Control Area (Grevena)	Sample Area (Kozani)	Test Statistic (U)	Z-Value	*p*-Value
d-ROMs (U.CARR)	569.62 ± 22.52	630.22 ± 33.76	1098.00	−1.048	0.295

Note: d-ROMS: Derivatives of reactive oxygen metabolites.

**Table 3:** Comparison of serum BAP levels between pregnant women in control (Grevena) and polluted (Kozani) areas using the Mann–Whitney U test.

Parameter	Control Area (Grevena)	Sample Area (Kozani)	Test Statistic (U)	Z-Value	*p*-Value
BAP (μmol/L)	1996.54 ± 68.87	1500.18 ± 60.38	522.00	−5.019	0.000

Note: ΒAΡ: biological antioxidant potential.

**Table 4:** ANCOVA results for d-ROMs and BAP levels by region after adjustment for covariates.

Dependent Variable	Group	Adjusted Mean ± SE	95% CI	F (df)	*p*-Value	Partial η^2^
d-ROMs (U.CARR)	Control	559.12 ± 26.75	506.01–612.23	4.52 (1.93)	0.035 *	0.046
	Sample	640.72 ± 26.75	587.61–693.83			
BAP (μmol/L)	Control	1996.29 ± 62.31	1872.56–2120.01	31.03 (1.93)	<0.001 *	0.250
	Sample	1500.43 ± 62.31	1376.71–1624.16			

Note: Adjusted for body mass index (BMI), years of residence, gestational age, weight at the beginning of pregnancy, and current weight. d-ROMs: derivatives of reactive oxygen metabolites; BAP: biological antioxidant potential; df: degrees of freedom; SE: standard error; CI: confidence interval; Partial η^2^: partial eta squared. Asterisks (*) indicate statistically significant differences between groups.

## Data Availability

The original contributions presented in this study are included in the article. Further inquiries can be directed to the corresponding author.
